# Motivation and Perceived Learning of Secondary Education History Students. Analysis of a Programme on Initial Teacher Training

**DOI:** 10.3389/fpsyg.2021.661780

**Published:** 2021-07-01

**Authors:** Cosme J. Gómez Carrasco, Jairo Rodríguez-Medina, Pedro Miralles-Martínez, Ramón López-Facal

**Affiliations:** ^1^CEIR Campus Mare Nostrum, University of Murcia, Murcia, Spain; ^2^Facultad de Educación, Universidad Nacional de Educación a Distancia, Madrid, Spain; ^3^Department of Applied Didactics, Education Faculty, University of Santiago de Compostela, Santiago de Compostela, Spain

**Keywords:** history education, teacher training, secondary education, motivation, perceived learning

## Abstract

This paper presents the results of research on the initial teacher training in Spain. The aim is to verify whether the development of a training program for teachers based on methodological and epistemological aspects has a positive repercussion on the learning of secondary school pupils. We evaluated to what degree this training was reflected in secondary education pupils (*n* = 467, M_age_ = 14.74, SD = 1.97) taught by the participating trainee teachers during their period of teaching practice. A pretest and a postest were employed to obtain data on the methodology and the motivation and perceived learning on the part of the pupils. A confirmatory factor analysis was carried out to verify the validity and internal consistency of the tools and, later, the longitudinal invariance in each of the dimensions analyzed. The results confirm the internal consistency and validity of the tools employed and the improvement in the pupils' evaluation regarding methodology, motivation, and learning.

## Motivation and Perceived Learning of Secondary Education History Students. Analysis of a Programme on Initial Teacher Training

In history classes, secondary school teachers and pupils do not usually discuss the practices of teaching and learning based on routines, neither do they question their relevance or effectiveness (VanSledright, 2014). These practices normally motivate teachers to prioritize certain instructional goals above those of other kinds (Voet and De Wever, 2020). Pupils, especial those who achieve greater academic success, adopt the same strategies, which are those which are socially accepted, to read, memorize, think, and write as they are required to (Nokes, 2017). Routines are reproduced and reinforced with the activities proposed in history classes, with the normal interaction with the teacher and the procedures and techniques of assessment (Gómez et al., 2020a). These routines are rooted in two sets of conceptions, one of which is epistemological and the other methodological.

For several decades, researchers in the field of history education have debated about how historical contents can be adapted to the learner, although greater emphasis has generally been placed on the transformation of epistemological conceptions than on the practice of teaching in the classroom. Thus, the works of Monte-Sano (2011), Reisman (2012), and Van Boxtel and Van Drie (2012), are related with issues such as historical thinking, historical literacy, and the use of primary sources in the classroom. From another standpoint, Carretero and Van Alphen (2014), Grever et al. (2011), López et al. (2014), and Wilschut (2010) have focused on issues of historical consciousness, identity, and historical memory.

Recent monographs on the topic of history education have shown the increase in research in this field, particularly regarding the changing of the conceptual model of history teaching (Carretero et al., 2017; Metzger and Harris, 2018; Gómez et al., 2020b). Some work, such as that being carried out in the Netherlands, focuses on evaluative research which is more centered on the practice of teaching (De Groot-Reuvekamp et al., 2018; Van Straaten et al., 2018).

All the above aim to reformulate the role of pupils in the classroom, based on the assumption that learning history does not simply consist of memorizing a canonical narrative (Monte-Sano et al., 2014). Rather, it supposes a different kind of cognitive work which makes it possible to construct interpretations of the past based on sources and tests (VanSledright, 2014; Nokes, 2017), a work which implies the involvement of the learner in the techniques of the analysis of the past. Unlike what normally happens in the classroom, where texts are used to transmit information which pupils must memorize, historians interpret documents as evidence to build explanations for historical processes (Lesh, 2011). The method of the historian is a key factor in the conception of history as a science, it consists of developing the capacity to interrogate a historical source, to contextualize it and seek answers.

To improve history education, it is necessary for teachers to incorporate teaching methods which diverge from traditional approaches, accompanied by an epistemological change (Wineburg, 2001).

### The Present Study

Tuithof et al. (2019) recently carried out a systematic analysis of research on PCK in history education. They highlight the large number of qualitative studies with small samples and point out that a large proportion of these studies deal with disciplinary strategies of history such as argumentation and the use of primary sources in the classroom (Burn, 2007; Van Hover and Yeager, 2007; Monte-Sano and Budano, 2013; Ledman, 2015). There is a relative lack of studies which focus on issues of educational methodology or on pedagogical strategies and, when they do so, they approach the issue from some specific aspect of the subject such as the teaching of controversial topics and critical pedagogy in the classroom (Blevins et al., 2020). Most research on the evaluation of training programmes has based its results on the analysis of exercises developed by teachers, on direct observation or on perception questionnaires (De Groot-Reuvekamp et al., 2018; Miralles et al., 2019a,b). There are also very few studies which have evaluated the improvement of competences of initial trainee teachers when they start their teaching practice, verifying the effectiveness of the programmed activities. Indeed, when this has been done, it has been *via* small samples and employing qualitative techniques (Gómez and Miralles, 2017).

Faced with a scarcity of this type of study, in this paper we propose the following hypothesis: the implementation of an intervention programme in teacher training based on methodological and epistemological elements of the didactic knowledge of historical knowledge contributes toward optimizing teaching competences and improving pupils' learning. To verify this hypothesis, the aim was proposed of analyzing the effects of this intervention programme through the changes perceived by the pupils in terms of their motivation and learning. The evaluation of the programme has been carried out *via* the implementation of the teaching units designed by the trainee teachers and verifying, when putting them into practice, their effect on pupils in the secondary school classroom. To evaluate these changes, indicators of motivation and perceived learning have been employed (Makransky and Lilleholt, 2018; Deslauriers et al., 2019; Maloy et al., 2019). To achieve this general objective, the following specific objectives and two hypotheses have been proposed:

1. To analyse the factorial longitudinal invariance of the subscales of motivation and perceived learning both before and after the implementation of the intervention programme.
2. To identify the changes brought about in the secondary school pupils studying history as far as their motivation and perceived learning are concerned following the intervention programme.
3. To differentiate the changes in motivation and perceived learning among the pupils according to the intensity of the intervention.

H1: Student motivation is significantly higher in courses with high levels of intensity of the intervention than those with low levels of intensity.
H2: Student perceived learning is significantly higher in courses with high levels of intensity of the intervention than those with low levels of intensity.

## Method

### Design

An exploratory study was carried out with a design of multiple independent groups in which the intervention was applied with three levels of intensity. As pointed out by Arnau and Balluerka (2004), multilevel linear models for longitudinal data are an appropriate tool for the evaluation of interventions in the field of the behavioral sciences.

### Participants

Four hundred and sixty-seven pupils from 18 secondary education classes took part in the research. These 18 classes came from 14 different schools (13 state-run and one private). The teaching units were put into practice in the 4 years of compulsory secondary education (ESO: 12–16 years of age) and in the 2 years of Baccalaureate (16–18 years of age). Six pupils were eliminated from the research for having completed less than a third of the items. The selection of the sample was related with the assignation of schools for the teaching practice of the trainee teachers who were going to implement the teaching units. The percentage of participants according to sex is similar, although there were slightly more girls than boys (Female = 245, 52.46%; Male = 222, 47.54%). Although data was gathered for all the years of secondary education, 31.12% of the sample was from the 1st year (150), 17.34% from 2nd year (81), 4.07% from 3rd year (19), 26.77% from 4th year (125), 16.27% from 1st Baccalaureate (76), and 3.43% from 2nd Baccalaureate (16). A statistical power analysis was performed for sample size estimation. With an alpha = 0.05 and power = 0.80, the projected sample size needed is ~*N* = 120 to detect medium effects (η^2^ = 0.09).

### Procedure

#### Design of the Intervention Programme

An intervention programme was designed in the subject “Methods and resources for the teaching of geography, history and the history of art” in the geography and history speciality of a master's degree in Teacher Training. The main objective was to improve the skills of the future teachers in the design of activities and teaching units. In this programme, epistemological elements (emphasizing the competences of historical thinking) were combined with methodological elements (active teaching strategies, research methods, digital resources, etc.). The aim was that the trainee teachers would modify their methodological approach (how to teach history).

All procedures were in accordance with the ethical standards of the institutional and national research committee and with the 1964 Helsinki declaration and its later amendments or comparable ethical standards and was approved by the ethics committee of the university.

### Intervention

The formative programme consisted of eight sessions of 4 h each. The first three were devoted to working on active learning methods: project method, case studies, problem-based learning, simulations, gamification and flipped classroom (Table 1). The first session was dedicated to answering the question “Why is a change in the teaching model for geography and history necessary?” and was linked to the work the students had already carried out in previous subjects regarding historical competences. In the second and third sessions it was specifically these research methods that were developed and exemplified. The following two sessions were devoted to working with primary sources, heritage, and digital resources. In these sessions, work on proposals for methodological change was combined with examples which developed these proposals using historical thinking skills. The final three sessions were used to construct the teaching units, applying the theoretical work to the teaching unit which they would later put into practice with secondary school pupils. Eighteen of the trainee teachers participated in the evaluation of these teaching units in their period of teaching practice.

**Table 1:** Definition of the sessions of the formative programme.

**Session**	**Description**
Session 1	Why is a change in the teaching model for geography and history necessary? Analysis of diagnostic and comparative research with other territorial realities: England and Canada. Influence on epistemological aspects (the six historical thinking skills proposed by Seixas) for a change in the teaching model.
Session 2	Research strategies (I). Influence on research work with pupils (searching for, selecting, and analyzing information). Work on collaborative techniques such as Aronson's jigsaw technique, the use of classroom debates, communicative strategies, Project/Problem-Based Learning, case studies, and Service-Learning. Presentation and debate on specific practical examples.
Session 3	Research strategies (II). Simulation strategies such as the use of drama and historical perspective; flipped classroom, gamification, and fieldwork *via* experimental educational trips. Presentation and debate on specific practical examples.
Session 4	Primary sources and heritage. Analysis of the usefulness of primary sources in the classroom, work on studies on heritage education, examples of typology of heritage assets, guided work on where to find primary sources on the Internet and how to include them in the teaching units. Specific work on the official website of the Spanish archives (PARES).
Session 5	Digital resources. Digital competence, online resources, general applications (such as WebQuest, Wikis and Blog) and specific applications of use for geography and history (Google Earth, National Geographic's MapMaker Interactive, virtual museums, virtual recreations, etc.). Work on Kahoot and Socrative to introduce gamification.
Session 6	Curricular framework for the teaching unit. Objectives, contents, and competences.
Session 7	Methodology and activities. Methodological explanation and sequence of activities: initiation-motivation, introduction of knowledge, synthesis, and application.
Session 8	Evaluation. Procedure, techniques, and tools of evaluation. What, who, how and when to evaluate?

To ensure the fidelity of the intervention, a checklist was created with the 12 strategies and techniques which had been worked on in the intervention programme. Six of these strategies are methodological in character: research activities, collaborative techniques, discussions and debates, digital resources, use of portfolios and classwork for evaluation and the use of direct observation, rubrics, and observation scales. The other six techniques and strategies are of an epistemological nature, related with the historical competences put forward by Seixas and Morton (2013): historical significance, work on historical sources, causes and consequences, continuity and change, historical perspectives, and the ethical dimension of history. As far as the methodological variables are concerned, the majority used digital resources, discussions and debates and the use of portfolios and classwork. Collaborative techniques were employed by two thirds of the participating teachers, whereas research activities were used in half of the interventions. Direct observation was only used in four out of 10 cases (Table 2). As for the epistemological variables, most of the interventions made use of work with sources, and activities regarding cause and consequence and continuity and change. Activities on the ethical dimension of history were used in a little more than half of the teaching units. On the other hand, activities regarding historical perspectives and historical significance had less presence.

**Table 2:** Methodological and epistemological variables introduced in the teaching units designed by the master's students.

**Methodological variables**	**Number**	**Percentage**
Digital resources	17	94.44
Discussions and debates	17	94.44
Use of portfolios and classwork	17	94.44
Collaborative techniques	12	66.67
Research activities	9	50.00
Direct observation, rubrics and observation scales	7	38.89
**Epistemological variables**	**Number**	**Percentage**
Activities on change/continuity	16	88.89
Use of historical documents	15	83.33
Activities on causes/consequences	13	72.22
Activities on ethical dimension	10	55.56
Activities on historical perspectives	3	16.67
Activities on historical significance	3	16.67

### Instruments

To evaluate the implementation of the teaching units, two tools were designed, one pretest and one postest. In this study, the data from these tools referring to three categories (methodology, motivation, and perceived learning) were collected. The pretest and postest items were the same. While the pretest evaluated the history classes which the pupils had received up to the starting date, the postest evaluated the implementation of the teaching unit designed by the trainee teachers. The validation of the content was carried out *via* the interjudge procedure regarding categories of relevance and the clarity of the items of the tool. For this content validation, the decision was made to create a discussion group with seven experts: two professors from the field of social science education, two secondary education geography and history teachers, two primary education social science teachers and a professor from the Department of Research and Diagnostic Methods in Education, an expert in research methodology. The decision was taken to use the Delphi method and, following the relevant modifications, a second round was carried out with the experts to definitively validate the two tools (Gómez et al., 2020c).

The internal consistency of the three subscales and the total scoring was proved *via* Cronbach's alpha coefficients for ordinal data (Gadermann et al., 2012) and McDonald's omega (Revelle and Zinbarg, 2009; McDonald, 2013). Overall, ordinal alpha values of 0.92 in the pretest and 0.92 in the postest were obtained, along with total reliability coefficients of 0.91 in the pretest and 0.92 in the postest. Both values were considered excellent. As far as the first subscale, relating to methodology, is concerned, acceptable rates of internal consistency were obtained (pretest α = 0.70, ω = 0.78; postest α = 0.72, ω = 0.77). As for the second subscale, relating to the pupils' motivation, indices of reliability which can be adequate were obtained (pretest α = 0.89, ω = 0.90; postest α = 0.89, ω = 0.92). The third subscale, regarding the pupils' perception of learning, obtained good indices of reliability (pretest α = 0.90, ω = 0.91; postest α = 0.89, ω = 0.9).

### Data Analysis

**Figure 1:**
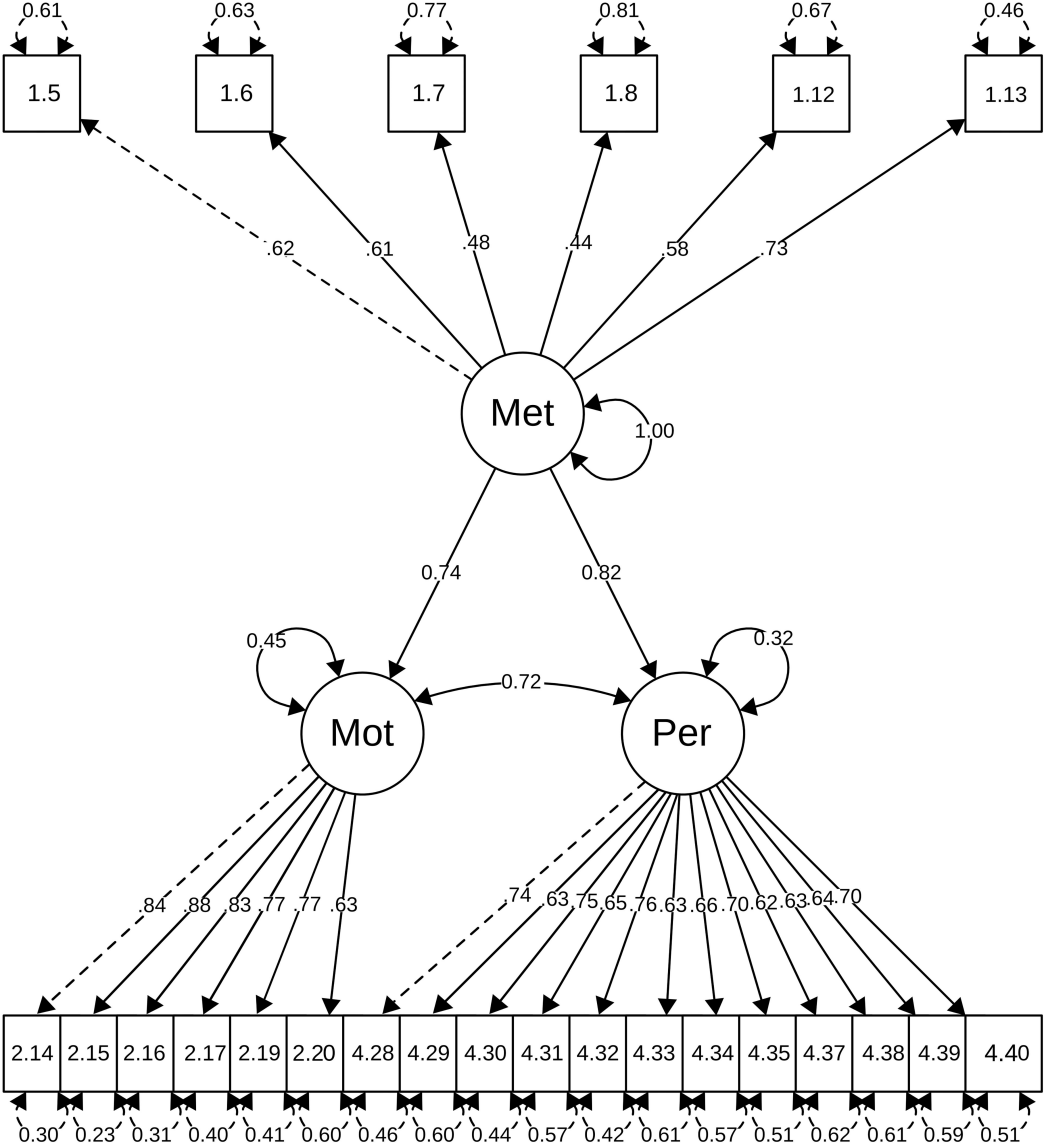
Structural model results.

To examine construct validity, we carried out structural equation modeling (SEM) to confirm the existence of a series of constructs in the questionnaire. All models were estimated by weighted least squares WLSMV on the polychoric correlations matrix (Hair et al., 2010). Goodness-of-fit was checked using the comparative fit index (CFI) and Tucker-Lewis index (TLI) and the root mean square error of approximation (RMSEA). Figure 1 shows the definition of the structural equation model, in which the two-way arrows represent the covariances between the latent variables (ellipses) and the one-way arrows symbolize the influence of each latent variable (constructs) on their respective observed variables (items). The standardized estimates of the path coefficients for each variable are also shown. Lastly, the two-way arrows over the squares (items) show the error associated to each observed variable.

The analytical strategy was carried out in three phases. In the first of these, after proving the suitability of the data for factor analysis, the longitudinal factorial invariance was verified. To do this, a succession of models was specified for each scale, with each being more restrictive than the previous model. The factorial invariance between the pretest and postest was progressively analyzed. The progressive estimation of the invariance begins with the baseline model (configural invariance) and continues with the invariance levels of factor loadings (metric), of thresholds (strong invariance) and, finally, of strict invariance (Liu et al., 2017). The procedure proposed by Liu et al. (2017) was followed for checking the longitudinal invariance with ordinal data and to evaluate the practical relevance of the invariance violation by way of sensitivity analysis.

As far as the analysis of the longitudinal invariance is concerned, four levels of invariance were analyzed: configural, factor loadings (metric), thresholds (strong) and strict. The first of these is the invariance in the basic configuration of the measurement model. In this case, the reference model proves the hypothesis that the same general pattern of factor loadings stays constant over time. To check the second level of invariance, the previous model was compared with the factor loading invariance model. This model adds the restriction that the factor loadings are identical over time. Then, this invariance model of factor loadings was compared with the invariance model of thresholds. This model adds the restriction that, for each indicator, the thresholds are invariant on the two occasions on which they are measured.

As far as the sensitivity analysis is concerned, to check the practical relevance of the supposed longitudinal invariance violation, the analysis of probabilities proposed by Liu et al. (2017) makes it possible to compare the probabilities of selecting each one of the response options which predict models with different levels of invariance. The differences in the probabilities which predict the models can serve as an estimate of the size of the effect of the longitudinal invariance violation (Liu et al., 2017). These probabilities are estimates of the percentage of those surveyed who select each category of response, in each item and on each occasion (pretest—postest), supposing a specific model of invariance. The fit of the different structures obtained was compared by way of confirmatory factor analysis. All the models were estimated *via* “WLSMV” weighted least squares. The goodness of fit was contrasted by using comparative fit (CFI) and Tucker-Lewis (TLI) indices and the root mean square error of approximation (RMSEA). It was considered that CFI and TLI indices higher than 0.90 indicate acceptable degrees of fit and that above 0.95 is good (Hu and Bentler, 1999). As far as the RMSEA is concerned, values the same or lower than 0.05 were interpreted as good and <0.08 as acceptable (Browne and Cudeck, 1992; Hu and Bentler, 1999). The recommendations of Chen (2007) and Cheung and Rensvold (2002) were followed, according to which increases of <0.010 in CFI and TLI and decreases of <0.015 in RMSEA suggest that there are no relevant changes in the fit of a model regarding the following, more restrictive, one, in establishing the relevance of the differences in fit between models.

In the third phase, the factor scores obtained for motivation and perception of learning were compared according to the intensity of the application of the intervention, for which the multivariate analysis of variance (MANOVA) was used.

All the analyses were carried out using the *lavaan* package (Rosseel, 2012) in the free statistical software R 3.6.3 (R Core Team, 2020) and Mplus 7.0 (Muthén and Muthén, 2015).

## Results

The results of the SEM analysis of the hypothetical model demonstrated satisfactory fit indexes: RMSEA = 0.050, SRMR = 0.062, CFI = 0.991, and TLI = 0.990. The composite reliability (CR) of each latent variable was calculated. Values of CR = 0.751 (Methodology); CR = 0.908 (Motivation), and CR = 0.909 (Perceived learning) were obtained. Since in all cases the values of CR are higher than 0.70, we can conclude that the indicators of the three subscales, considered together, are a reliable measure of the construct (Hair et al., 2010). There is evidence of convergent validity if the items that are indicators of a specific construct share a high proportion of the common variance. This is confirmed in the present case, since (a) the saturations of all the items have been statistically significant; (b) only two of them showed values lower than 0.5 (item 1.7 = 0.48; item 1.8 = 0.44).

### Longitudinal Invariance

To investigate the longitudinal factorial invariance and the change in each one of the latent variables, the correlation matrix observed between the indicators of each one of the first-order factors was examined with the aim of checking whether, in general, the model fitted the structure of the data (Wickrama et al., 2016; Grimm et al., 2017). Little (2013) suggests that a longitudinal confirmatory factor analysis (LCFA) model is appropriate when the correlation coefficients between the indicators of the global latent variable at the same point in time are higher than the correlation coefficients between the same indicators at different points in time. It was observed that the correlations at each of the moments of data collection tended to be stronger than the correlations between the different moments. The correlations between the six indicators of the motivation variable (items 2.14–2.21) are higher at the same moment in time and lower at different moments of data collection. More specifically, these correlation coefficients varied between 0.37 and 0.78 in the pretest and 0.37 and 0.76 in the postest, while the correlations at the different points in time were notably lower, ranging from 0.02 to 0.20 for the variable of motivation. The correlations between the indicators of the variable *perceived learning* (items 4.28–4.40) were higher at the same point in time and lower at different moments of data collection. More specifically, these correlation coefficients varied between 0.19 and 0.75 in the pretest and 0.06 and 0.54 in the postest, while the correlations between the different points in time were notably lower, ranging from 0.01 to 0.21.

For the specification of the models of longitudinal invariance in the subscale of motivation, item 2.15 (“The history classes improve my motivation to learn and to make more of an effort”) was selected as the marker item as it showed a high factor loading in both the pretest (λ_2_ = 0.912) and the postest (λ_2_ = 0.907), which did not differ between testing moments. Table 3 presents the fit indices for each level of longitudinal factorial invariance in the subscale of motivation. The hypothesis of configural invariance (the baseline model for ordinal data) is accepted as the six items loaded positively in just one factor at each moment of measurement. The fit indices show a suitable fit for the model, thereby supporting the supposition that these items represent the same underlying construct at each point of time. The results did not support the hypothesis of metric invariance (loading invariance—scale invariance) as a significant increase of the Δχ^2^ difference (*p* = 0.028) was caused. However, no change was observed in the comparative fit index CFI (ΔCFI = 0) regarding the model of configural invariance. The model of threshold (strong) invariance of the data also produced a significant Δχ^2^ (*p* < 0.05) between the metric (scale) invariance but only a slight change in the CFI (ΔCFI <0.01). Last of all, strict invariance was examined. The model of strict invariance increased the χ^2^ goodness of fit test as was to be expected. The results showed a significant Δχ^2^ (*p* < 0.05). Yet again, no notable change in the CFI was found (ΔCFI < 0.01).

**Table 3:** Analysis of the longitudinal factorial invariance for the motivation variable.

**Motivation**	**Configural invariance**	**Metric invariance**	**Strong invariance**	**Strict invariance**
	**(Baseline model)**	**(Loading)**	**(Threshold)**	**(Unique)**
χ^2^ / *df* fit	82.860 / 47	91.470 / 52	167.806 / 69	214.664 / 75
	115.383 / 47	126.112 / 52	219.328 / 69	249.374 / 75
*p* χ^2^	0.001 – 0	0.001 – 0	0	0
Δχ^2^/Δ *df* fit	-	9 / 5*	76 / 17***	47 / 6**
CFI	0.998 – 0.992	0.998 – 0.991	0.995 – 0.982	0.993 – 0.979
ΔCFI	-	0 - 0	0.003 – 0.009	0.002 – 0.003
TLI	0.997 – 0.989	0.997 – 0.989	0.995 – 0.983	0.993 – 0.982
RMSEA (CI)	0.040 (0.026 – 0.054)	0.040 (0.026 – 0.054)	0.055 (0.045 – 0.066)	0.063 (0.053 – 0.073)
	0.056 (0.043 – 0.069)	0.055 (0.043 – 0.068)	0.068 (0.058 – 0.078)	0.070 (0.061 – 0.080)
*p* ≤ 0.05	0.863–0.162	0.878 – 0.231	0.196 – 0.002	0.015 – 0
SRMR	0.045 – 0.045	0.046	0.046	0.048

*CFI, comparative fit index; df, degrees of freedom; RMSEA, root mean square error of approximation; SRMR, standardized root mean square residual; TLI, Tucker–Lewis index; CI, confidence interval*.

An asterisk (*) next to the Δχ^2^ indicates that the model was statistically significant from the previously specified model.

These results indicate the necessity to ascertain to what degree the violation of the supposed strict longitudinal invariance could affect the interpretation of the second-order growth model for the motivation scale. It is necessary, therefore, to investigate the practical relevance of the violation of this supposition. More specifically, it should be investigated when (that is to say, at which moment of the test) and where (in which item and category of response) the infraction has a substantial impact and to what degree the changes in the scores between the pretest and postest are affected. To do this, a sensitivity analysis was carried out as proposed by Liu et al. (2017), the results of which are presented in Table 4, which shows the probabilities of each option being chosen which predict the metric (loading) invariance and strong (threshold) invariance for each indicator at each moment of measurement and makes it possible to verify the discrepancies in the probabilities predicted between both models. The differences in the predicted probabilities between these two models may serve as an estimation of the size of the effect of the longitudinal invariance violation (Liu et al., 2017). The threshold (strong) invariance violation produced small differences in the probabilities of selection of each response option. As can be seen, the biggest discrepancy in the pretest was 0.058 (0.236 – 0.178) which occurred in response option 5 of variable 2.21 (“I am motivated in the history classes because we use resources other than the textbook: Internet, audio-visual resources, historical documents”, etc.). As far as the postest is concerned, the biggest discrepancy occurred again in response option 5 of variable 2.21 (diff = 0.063; 0.449 – 0.38). Liu et al. (2017) do not suggest a specific cut-off value to interpret this difference, although they do point out that differences of <0.05 should not noticeably affect the estimation of the parameters of the second-order growth model. Overall, these results indicate that the rejection of the threshold invariance does not substantially affect the choice of a specific response category for a specific item administered at one moment of a specific test. Therefore, in this case, the changes between the pretest and the postest in the expected means, the variances and covariances would be wholly attributable to the changes in the common latent factor over time.

**Table 4:** Estimated probabilities for the metric invariance models (scale invariance—loading invariance model) and strong invariance (threshold invariance).

**Indicator**	**Response option (degree of agreement)**				
	**Strongly disagree**				**Strongly agree**
**Pretest**					
Motivation 2.14	0.154 – 0.154	0.149 – 0.152	0.283 – 0.270	0.265 – 0.282	0.149 – 0.141
Motivation 2.15	0.137 – 0.149	0.197 – 0.187	0.277 – 0.291	0.292 – 0.273	0.096 – 0.100
Motivation 2.16	0.111 – 0.120	0.173 – 0.163	0.217 – 0.262	0.332 – 0.309	0.167 – 0.145
Motivation 2.17	0.126 – 0.130	0.172 – 0.170	0.269 – 0.297	0.268 – 0.254	0.165 – 0.149
Motivation 2.19	0.202 – 0.179	0.165 – 0.191	0.330 – 0.315	0.208 – 0.191	0.094 – 0.123
Motivation 2.21	0.141 – 0.126	0.136 – 0.142	0.254 – 0.210	0.291 – 0.286	**0.177 – 0.235**
**Postest**					
Motivation 2.14	0.035 – 0.032	0.065 – 0.059	0.182 – 0.180	0.373 – 0.342	0.344 – 0.387
Motivation 2.15	0.031 – 0.021	0.069 – 070	0.227 – 0.224	0.415 – 0.409	0.258 – 0.275
Motivation 2.16	0.022 – 0.017	0.059 – 0.058	0.235 – 0.183	0.413 – 0.410	0.271 – 0.333
Motivation 2.17	0.026 – 0.024	0.075 – 0.069	0.263 – 0.219	0.353 – 0.334	0.283 – 0.353
Motivation 2.19	0.020 – 0.036	0.129 – 0.101	0.320 – 0.323	0.294 – 0.312	0.237 – 0.227
Motivation 2.21	0.022 – 0.027	0.084 – 0.072	0.122 – 171	0.323 – 0.345	**0.449 – 38**

*The values in bold are those representing discrepancies >0.05 in absolute value*.

For the specification of the longitudinal invariance models in the subscale of perceived learning, item 4.30 (“In the history classes I learn to use chronology”) was selected as the marker item as it demonstrated a high factor loading in both the pretest (λ_2_ = 0.738) and the postest (λ_2_ = 0.708), which did not differ between moments of testing. Table 5 presents the fit indices for each level of longitudinal factorial invariance in this subscale. It can be observed that the hypothesis of configural invariance (baseline model for ordinal data) is accepted as the 11 items loaded positively in only one factor at each moment of measurement. The fit indices showed an adequate fit for the model, which supports the supposition that these items represent the same underlying construct at each point in time. The results do not support the hypothesis of metric invariance (loading invariance—scale invariance) because there was a significant increase in the Δχ^2^ difference (*p* < 0.001). However, there was a change in the comparative fit index CFI (ΔCFI = 0.001), meaning that the fit of the model cannot be considered significantly worse regarding the configural invariance model. The threshold (strong) invariance model of the data also produced a significant Δχ^2^ (*p* < 0.05) between the metric (scale) invariance, although there was, again, only a slight reduction in the CFI (ΔCFI = 0.004). Last of all, strict invariance was examined. The model of strict invariance increased the χ^2^ goodness of fit test, as was to be expected. The results showed a significant Δχ^2^ (*p* < 0.05) and, again, no notable change in the CFI was found (ΔCFI < 0.01).

**Table 5:** Analysis of longitudinal factorial invariance for the variable perceived learning.

	**Configural invariance**	**Metric invariance**	**Strong invariance**	**Strict invariance**
	**(Baseline model)**	**(Loading)**	**(Threshold)**	**(Unique)**
**Perce. Learning**				
χ^2^ / *df* fit Standard Robust	572.700 / 198	600.182 / 208	715.103 / 239	867.715 / 250
	566.669 / 198	590.734 / 208	695.206 / 239	776.392 / 250
*p* χ^2^	0 – 0	0 – 0	0 – 0	0 – 0
Δχ^2^/Δ *df* fit	–	28 / 10*	115 / 31*	152 / 11
CFI	0.982 – 0.950	0.981 – 0.948	0.977 – 0.938	0.970 – 0.929
ΔCFI		0.001 – 0.002	0.004 – 0.01	0.007 – 0.009
TLI	0.979 – 0.942	0.979 – 0.943	0.978 – 0.940	0.972 – 0.934
RMSEA (CI)	0.064 (0.058 – 0.070)	0.064 (0.058 – 0.070)	0.065 (0.060 – 0.071)	0.073 (0.067 – 0.078)
	0.063 (0.057 – 0.069)	0.063 (0.057 – 0.069)	0.064 (0.058 – 0.069)	0.067 (0.062 – 0.073)
*p* ≤ 0.05	0 – 0	0 – 0	0 – 0	0 – 0
SRMR	0.065	0.066	0.066	0.069

*CFI, comparative fit index; df, degrees of freedom; RMSEA, root mean square error of approximation; SRMR, standardized root mean square residual; TLI, Tucker–Lewis index; CI, confidence interval*.

An asterisk (*) next to the Δχ^2^ indicates that the model was statistically significant from the previously specified model.

These results oblige us to ascertain to what degree the supposed threshold invariance violation could bias the estimation of the parameters of the growth model. Therefore, the practical relevance of the violation of this supposition should be investigated. Specifically, it should be investigated when (that is to say, at what moment of the test) and where (in which item and response category) the infraction has a substantial impact, and to what degree the changes in the scores between the pretest and postest are affected. To achieve this, a sensitivity analysis was carried out as proposed by Liu et al. (2017), the results of which are presented in Table 6, which shows the probabilities of the election of each response option predicting the metric (loading) invariance and strong (threshold) invariance models for each indicator at each moment of measurement and makes it possible to verify the discrepancies in the predicted probabilities between both models. The differences in the predicted probabilities between these two models may serve as an estimate of the size of the effect of the longitudinal invariance violation (Liu et al., 2017). The threshold (strong) invariance violation produced small differences in the probabilities of selection for each of the response options. As can be observed, only two of the 110 differences were slightly higher than 0.05 in the pretest and in the postest. In particular, the percentage of pupils which the model predicted would choose response option 5 in the pretest for item 4.31 (“In the history classes I learn to handle documents and historical sources”) was 6.2% higher (0.134 – 0.072 = 0.062) in the threshold invariance model than in the metric (factor loading) model. However, in the postest, the percentage of pupils which the model predicted would choose response option 5 for item 4.29 (“In the history classes I learn about the main historical figures”) was 6.4% higher (0.375 – 0.311 = 0.064) in the threshold invariance model than in the metric (factor loading) model. Liu et al. (2017) do not suggest a specific cut-off value for interpreting this difference, although they do point out that small differences in a few items and for a few response options should not noticeably affect the estimation of the parameters of the growth model. Therefore, the general results indicate that the rejection of threshold invariance does not substantially affect the election of each of the specific response options for each item administered on each occasion. In this way and in this case, the changes between the pretest and the postest in the expected means, the variances and the covariances would also be completely attributable to the changes in the common latent factor over time.

**Table 6:** Estimated probabilities for the metric invariance and strong invariance models.

**Indicator**	**Response options (degree of agreement)**									
	**Strongly disagree**		**Disagree**				**Agree**		**Strongly agree**	
**Pretest**	*Load*.	*Thres*.	*Load*.	*Thres*.	*Load*.	*Thres*.	*Load*.	*Thres*.	*Load*.	*Thres*.
Perceived L. 4.28	0.048	0.055	0.085	0.075	0.173	0.168	0.404	0.443	0.290	0.259
Perceived L. 4.29	0.039	0.040	0.092	0.088	0.201	0.227	0.439	0.460	0.229	0.184
Perceived L. 4.30	0.062	0.089	0.189	0.152	0.276	0.322	0.319	0.299	0.154	0.137
Perceived L. 4.31	0.118	0.113	0.149	0.143	0.362	0.315	0.299	0.295	**0.072**	**0.134**
Perceived L. 4.32	0.039	0.050	0.108	0.090	0.232	0.255	0.440	0.424	0.181	0.181
Perceived L. 4.33	0.089	0.090	0.129	0.125	0.321	0.320	0.288	0.297	0.173	0.168
Perceived L. 4.34	0.058	0.056	0.070	0.072	0.259	0.254	0.381	0.420	0.231	0.198
Perceived L. 4.35	0.060	0.064	0.115	0.108	0.232	0.233	0.369	0.392	0.224	0.203
Perceived L. 4.37	0.063	0.058	0.135	0.138	0.363	0.353	0.293	0.301	0.146	0.151
Perceived L. 4.39	0.126	0.119	0.166	0.167	0.307	0.263	0.252	0.280	0.149	0.171
Perceived L. 4.40	0.101	0.106	0.096	0.087	0.232	0.204	0.328	0.322	0.243	0.281
**Postest**										
Perceived L. 4.28	0.033	0.027	0.031	0.040	0.098	0.104	0.425	0.380	0.412	0.449
Perceived L. 4.29	0.033	0.032	0.052	0.056	0.177	0.146	0.428	0.391	**0.311**	**0.375**
Perceived L. 4.30	0.047	0.029	0.059	0.085	0.318	0.276	0.359	0.376	0.217	0.235
Perceived L. 4.31	0.033	0.037	0.081	0.088	0.254	0.295	0.386	0.383	0.246	0.197
Perceived L. 4.32	0.024	0.017	0.031	0.043	0.193	0.173	0.430	0.446	0.322	0.321
Perceived L. 4.33	0.031	0.031	0.070	0.073	0.277	0.278	0.371	0.363	0.251	0.255
Perceived L. 4.34	0.020	0.022	0.038	0.037	0.168	0.173	0.472	0.430	0.302	0.338
Perceived L. 4.35	0.024	0.021	0.050	0.056	0.171	0.170	0.449	0.425	0.305	0.328
Perceived L. 4.37	0.015	0.020	0.081	0.078	0.289	0.298	0.367	0.360	0.248	0.243
Perceived L. 4.39	0.028	0.033	0.105	0.104	0.223	0.261	0.397	0.374	0.247	0.227
Perceived L. 4.40	0.037	0.034	0.039	0.046	0.129	0.154	0.343	0.350	0.452	0.416

*The values in bold are those which represent discrepancies >0.05 in absolute value*.

### Differences Between the Postest and the Pretest and Intensity of the Intervention

Table 7 presents the means in the pretest and postest, the differences between the means and the results of the Wilcoxon signed-rank tests for each of the variables observed at the two moments of measurement. As can be observed, these statistics indicate a significant increase in the value of each item of the three subscales in the test carried out following the intervention. As far as the *methodology* variable is concerned, the mean effect size can be high (*Z* = 11.13; *p* < 0.001; *r* = 0.522), whereas regarding the *motivation* (*Z* = 9.4; *p* < 0.001; *r* = 0.443) and *perceived learning* (*Z* = 7.08; *p* < 0.001; *r* = 0.335) variables the mean effect size was moderate. In the methodology subscale, the rating of the use of research in the classroom stands out, along with critical work on historical events, the use of drama and the use of digital resources by teachers. As far as the motivation subscale is concerned, the evaluation made by the pupils regarding the improvement in their motivation to learn and apply themselves and their motivation to learn more about history stands out. In the case of the perceived learning subscale, the pupils' evaluation of their learning regarding group work, the use of digital resources, the capacity to interpret documents and primary historical sources and their capacity to debate current affairs stands out.

**Table 7:** Differences of averages between pretest-posttest in the evaluation of methodology.

**Items**	**Pretest**	**Postest**				**Effect size**
**Methodology**	**M**	**M**	**Diff**.	***Z***	***p***	***r***
Different tools are used for assessment (notebooks, written work, rubrics, portfolio, etc.)	3.62	4.15	0.53	7.46	<0.001	0.35
Historical documents are used in the classroom to learn history	2.69	3.68	0.99	10.74	<0.001	0.50
In history classes we use audio-visual resources (presentations, films, documentaries, etc.)	3.39	4.35	0.96	11.81	<0.001	0.555
In history classes we carry out research	2.51	3.64	1.13	12.67	<0.001	0.594
We put ourselves in the shoes of a historical figure (drama, simulations, etc.) in order to understand his/her actions	1.68	2.73	1.05	12.06	<0.001	0.567
We critique historical events and processes	2.65	3.49	0.84	12.06	<0.001	0.566
**Motivation**						
The classes motivate me to know more about history	3.11	3.92	0.81	10.12	<0.001	0.475
The history classes improve my motivation to learn and to make more of an effort	2.98	3.81	0.83	10.94	<0.001	0.516
The history classes motivate me because I gain a better understanding of the social and cultural reality with which I am in contact	3.29	3.85	0.56	7.54	<0.001	0.358
The history classes motivate me to achieve better marks	3.21	3.78	0.57	7.78	<0.001	0.365
I am motivated in the history classes because I can contribute my point of view and my own knowledge	2.82	3.68	0.86	10.01	<0.001	0.471
I am motivated in the history classes because we use resources other than the textbook (Internet, audio-visual resources, historical documents, etc.)	3.21	4.12	0.91	10.01	<0.001	0.471
**Perceived learning**						
In the history classes I learn about the main historical events	3.82	4.15	0.33	5.21	<0.001	0.245
In the history classes I learn about the main historical figures	3.74	3.92	0.18	2.80	=0.004	0.132
In the history classes I learn to use chronology	3.31	3.64	0.33	4.57	<0.001	0.215
In the history classes I learn to handle documents and historical sources	3.03	3.75	0.72	9.24	<0.001	0.435
In the history classes I learn about the changes and continuities of history	3.63	3.97	0.34	5.62	<0.001	0.264
In the history classes I learn that all historical figures and events are equally important	3.34	3.73	0.39	5.66	<0.001	0.266
In the history classes I learn about the causes and continuities of historical events	3.65	4.00	0.35	5.59	<0.001	0.263
In the history classes I learn about the reasons which led people in the past to act in a particular way and to critically evaluate their actions	3.60	3.94	0.34	5.55	<0.001	0.261
In the history classes I learn to carry out group work with my classmates	2.79	3.89	1.1	12.16	<0.001	0.576
In the history classes I learn to value more the heritage of our surrounding area	3.30	3.78	0.48	6.94	<0.001	0.329
In the history classes I learn about different ways of using IT for the teaching of the social sciences	2.68	3.67	0.99	11.35	<0.001	0.541
Thanks to the history classes, I am more respectful toward people of other cultures and with opinions which differ from my own	3.11	3.75	0.64	8.39	<0.001	0.403
The history classes help me to understand and debate current affairs	3.49	4.15	0.66	8.95	<0.001	0.429

**Figure 2:**
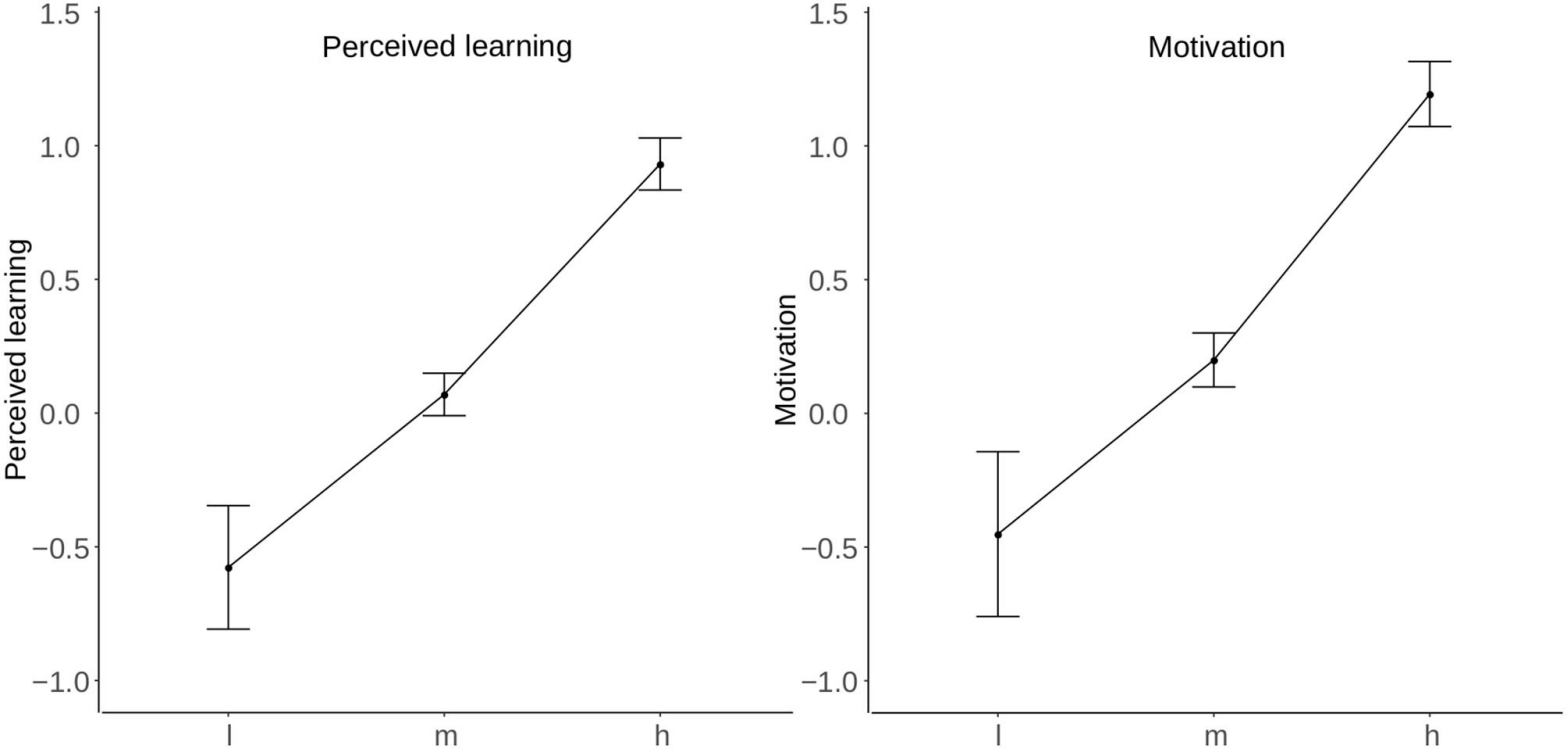
Difference of means according to the intensity of the intervention (l, low intensity; m, medium intensity; h, high intensity).

Finally, a one-way multivariate analysis of variance was carried out to compare the effect of the intervention on motivation and perceived learning in conditions of high, moderate, and low intensity of the intervention employing standardized factor scores (Figure 2).

The results showed a significant effect of the intensity of the intervention on motivation and perceived learning to the confidence level of 95% for the three conditions [Roy's Largest Root = 0.168, *F*_(4, 926)_ = 18.08, *p* < 0.001, η^2^ = 0.144].

Follow up univariate ANOVA's showed a significant effect of the intensity of the intervention on motivation to the confidence level of 95% for the three conditions [*F*_(2, 466)_ = 28.65, *p* < 0.001, η^2^ = 0.109]. The *post hoc* comparisons using Tukey's HSD test indicated that the mean score for the condition of high intensity of the intervention (M = 1.18, SD = 1.69) was significantly higher than the mean scores for the conditions of moderate (M = 0.194, SD = 1.4) and low intensity (M = −0.45, SD = 2.50). These results suggest that the high intensity intervention significantly increased the pupils' motivation compared to the moderate and low intensity interventions. Furthermore, the moderate intensity intervention also significantly increased the pupils' motivation compared to the low intensity intervention.

As far as perceived learning is concerned, the results also showed a significant effect of the intensity of the intervention of 95% for the three conditions [*F*_(2, 465)_ = 37.81, *p* < 0.001, η^2^ = 0.139]. The *post hoc* comparisons using Tukey's HSD test indicated that the mean score in perceived learning for the condition of high intensity of the intervention (M = 0.923, SD = 1.35) was significantly higher than the mean scores for the conditions of moderate (M = 0.069, SD = 1.12) and low intensity (M = −0.57, SD = 1.85). These results suggest that the high intensity intervention significantly increased the pupils' perceived learning compared to the moderate and low intensity interventions. Furthermore, the moderate intensity intervention also significantly increased the pupils' perceived learning compared to the low intensity intervention.

## Discussion

The results confirm the consistency of the tools used for data collection from the secondary school pupils. Strong correlations (longitudinal invariance) were observed between the indicators of the pretest and the postest, which make it possible to state that the secondary school pupils understand in the same way the questions asked before and after participating in the educational experiment: they have not modified the conception of what motivation and learning mean for them. The answers obtained, therefore, are relevant and significant to be able to validate the hypothesis.

On the other hand, the set of results relating to changes in the expected means, the variances and covariances, can only be attributed to changes in the common latent factor over time. The discrepancies between the pretest and the postest reflect the effects of the instruction process: it is probable that before the intervention many of the secondary pupils were unfamiliar with the active methodologies applied in this experimental phase. It should, therefore, be evaluated as a valid test of the positive effect of having incorporated methodologies in the motivation of the pupils, which, for them, were innovative.

The same occurs when evaluating the items related with perceived learning. All the items loaded positively in only one factor in both the pretest and in the postest. Therefore, it is confirmed that they represent the same underlying construct before and after the experiment. Thereby, in this case, the changes between the pretest and the postest in the expected means, the variances and the covariances would also be completely attributable to the changes in the common latent factor over time, in other words, to the effects of the teaching activities carried out in the classroom.

The pupils' evaluation of how they perceive teaching innovation has significantly improved. The schoolchildren gave great value to having participated in research in class, carrying out critical work on historical events, and the use of both drama and digital resources by teachers. That the pupils expressed their great satisfaction with the methodological change may explain why they perceive it as an improvement on the traditional teaching methodology, based on the transmission and reception of a master narrative, which is still widespread among history teachers today. Research's like Reisman and Enumah (2020), have improved the instruction using classroom video to help teachers identify curriculum-embedded opportunities for student discourse. This improved their understanding and facilitation of document-based historical discussions. De Leur et al. (2020) carried out an experimental study for the students to recreate historical situations through images and writing. The findings show that the written products contained more information elements than the drawings. However, in terms of the historical plausibility of the product, the drawn products and written products were comparable. Students who made a drawing reported higher situational interest than students who wrote a text. Studies such as those of Kavanagh et al. (2019) have shown the benefits of introducing discussion and debates in the classroom, both for the procedures of history and for the understanding of specific content. These international studies are showing the need to change the methodology in the classroom. But they also continue to show that the traditional methodology continues to survive.

Secondly, the pupils showed in the postest that they felt more motivated to learn history. This should be related not only with the changes in the way of working in the classroom but also with the changes in the contents introduced by the trainee teachers. As a result of this prior training, the trainee teachers designed teaching activities which prioritized the contents which they considered most appropriate for the education of their pupils as citizens, rather than for historical scholarship or the transmission of a master narrative. The responses of the secondary and baccalaureate pupils indicate that, in this way, they have managed to improve their effort and interest in historical knowledge.

An extremely positive, though slightly lower, evaluation was also achieved in relation to knowledge obtained because of collaborative work, the use of digital resources, the capacity of interpreting historical documents and primary sources and debates on current affairs.

Although the results are satisfactory for the three dimensions analyzed (methodology, motivation, and perceived learning), those relating to the methodology employed were higher, with those concerning perceived learning and motivation being more moderate, albeit also positive. This difference can be explained by the fact that changes in methodology are easily perceptible from the very first moment, whereas the perception of learning and the global evaluation of a subject requires much more time to be recognized. It is likely that a whole academic year would be necessary for this new approach to history education to be well-understood by the pupils.

It has been possible to demonstrate the significant effect of the intensity of the intervention on motivation and perceived learning. In both dimensions, the higher the intensity of the intervention on the part of the teachers, the better the perception and evaluation of the pupils have been. Although this effect may seem obvious, in research on history education the availability of tools for the observation of this phenomenon is not common. For this reason, as shown above, a one-way analysis of variance was carried out employing standardized factor scores.

Nevertheless, the research design does not allow isolating the novelty effect of the intervention program, so it would be desirable to check the effect of the methodology and the resources used when these are prolonged. Due to the design characteristics, with no control group, it is not possible to control the effects of history, maturation, and regression to the mean, which is a threat to internal validity. Furthermore, the analysis of the practical significance of the violation of different levels of invariance should be further investigated to confirm that it is equally sensitive to violations of different levels of invariance.

## Conclusions

Research on history education has, for decades, focused on identifying pupils' difficulties in learning about history and converting this knowledge into a useful tool for life (Nokes, 2017). There is a broad consensus on the importance of teachers modifying their teaching methodology and their epistemological conceptions so that their pupils can learn to employ historical thinking, or, in other words, to develop historical competences (Domínguez, 2015). It is clear that, to increase pupils' historical competence, it is necessary to improve the teaching skills of their teachers. There are few studies in the available literature on history education regarding the impact of the improvement of initial teacher training in the development of teachers' professional competence. The competence of teachers is reflected in the results of their pupils. The main contribution of this study is precisely this, to have evaluated to what degree a formative programme for teachers has had repercussions on their pupils: how the pupils perceive their own motivation and learning after their teachers have changed their methodology and the epistemological conceptions which modify and overcome traditional educational practices. It is shown empirically that the improvement in the teachers' training has had positive effects on the attitudes and learning of their pupils. One future line of research should be to analyse pupils' real learning, their historical competence, to interpret problems of the past and of the present: their capacity for relating these problems and developing their historical consciousness.

## Data Availability Statement

The raw data supporting the conclusions of this article will be made available by the authors, without undue reservation.

## Ethics Statement

The studies involving human participants were reviewed and approved by University of Murcia. Written informed consent to participate in this study was provided by the participants' legal guardian/next of kin.

## Author Contributions

CG: conceptualization, methodology, data curation, formal analysis, investigation, writing—original draft, and visualization. JR-M: conceptualization, methodology, data curation, formal analysis, writing—original draft, writing—review and editing, and visualization. PM-M: conceptualization, methodology, writing—review and editing, investigation, supervision, project administration, funding acquisition, and visualization. RL-F: conceptualization, methodology, investigation, writing—original draft, writing—review and editing, funding acquisition, and visualization. All authors contributed to the article and approved the submitted version.

## Conflict of Interest

The authors declare that the research was conducted in the absence of any commercial or financial relationships that could be construed as a potential conflict of interest.
